# The evolution of ^17^O-excess in surface water of the arid environment during recharge and evaporation

**DOI:** 10.1038/s41598-018-23151-6

**Published:** 2018-03-21

**Authors:** J. Surma, S. Assonov, D. Herwartz, C. Voigt, M. Staubwasser

**Affiliations:** 1 Institute of Geology and Mineralogy, University of Cologne, Cologne, Germany; 2 Present Address: International Atomic Energy Agency, Vienna, Austria

**Keywords:** Hydrology, Limnology, Palaeoclimate, Geochemistry, Hydrology

## Abstract

This study demonstrates the potential of triple O-isotopes to quantify evaporation with recharge on a salt lake from the Atacama Desert, Chile. An evaporative gradient was found in shallow ponds along a subsurface flow-path from a groundwater source. Total dissolved solids (TDS) increased by 177 g/l along with an increase in δ^18^O by 16.2‰ and in δD by 65‰. ^17^O-excess decreased by 79 per meg, d-excess by 55‰. Relative humidity (*h*), evaporation over inflow (*E*/*I*), the isotopic composition of vapor (^*^R_*V*_) and of inflowing water (^*^R_*WI*_) determine the isotope distribution in ^17^O-excess over δ^18^O along a well-defined evaporation curve as the classic Craig-Gordon model predicts. A complementary on-site simple (pan) evaporation experiment over a change in TDS, δ^18^O, and ^17^O-excess by 392 g/l, 25.0‰, and −130 per meg, respectively, was used to determine the effects of sluggish brine evaporation and of wind turbulence. These effects translate to uncertainty in *E*/*I* rather than *h*. The local composition of ^*^R_*V*_ relative to ^*^R_*WI*_ pre-determines the general ability to resolve changes in *h*. The triple O-isotope system is useful for quantitative hydrological balancing of lakes and for paleo-humidity reconstruction, particularly if complemented by D/H analysis.

## Introduction

Natural variations of δD and δ^18^O in H_2_O are widely used for evaporation studies and hydrological balancing of lakes^1–3^. Recent advances in mass-spectrometric analysis of the triple O-isotope system in water have shown that mass-dependent kinetic and equilibrium isotope fractionation between the liquid (l) and vapor (v) phase result in a measurably different fractionation coefficient,

1 $$\theta =\frac{\mathrm{ln}({}^{17}\alpha _{l-v})}{\mathrm{ln}({}^{18}\alpha _{l-v})},$$

with fractionation factors *α_*l*−*v_evap*_ for H_2_^17^O/H_2_^16^O and H_2_^18^O/H_2_^16^O^4,5^. In contrast to the classical D/H and ^18^O/^16^O system, *θ* calculated from ^17^O/^16^O and ^18^O/^16^O is relatively temperature insensitive because fractionation factors ^17^*α*_*l*−*v*_ and ^18^*α*_*l*−*v*_ change in a very similar fashion as functions of temperature^4,6^. Fractionation during evaporation of water (*α_*l−v_evap*_) is defined by *α for equilibrium exchange (*) and diffusion (*${\alpha }_{l-v{\rm{\_}}diff}$)^5,7^. The actual value of *θ* depends on the proportion of diffusion (*θ* = 0.5185) and equilibrium (*θ* = 0.529) isotope fractionation that a water sample has experienced. The proportion of diffusional fractionation depends on relative humidity (*h*):

2 $${}^{\ast }\alpha _{l-v\_evap}={}^{\ast }\alpha _{l-v\_eq}\cdot ({}^{\ast }\alpha _{l-v\_diff}\cdot (1-h)+h).$$

where * stands for 17 or 18, respectively. In isotope hydrology, variability in *θ* is reported as the ^17^O-excess parameter to visualize small deviations from the atmospheric compositional mean trend with respect to ocean water:

3 $${}^{{\rm{17}}}{\rm{O}} \mbox{-} \mathrm{excess}={\rm{\delta }}^{\prime} {}^{17}{\rm{O}}-0.528\cdot {\rm{\delta }}^{\prime} {}^{18}{\rm{O}},$$

where ${\rm{\delta }}^{\prime} {}^{17}{\rm{O}}=\,\mathrm{ln}({\rm{\delta }}{}^{17}{\rm{O}}+1)$ and ${\rm{\delta }}^{\prime} {}^{18}{\rm{O}}=\,\mathrm{ln}({\rm{\delta }}{}^{18}{\rm{O}}+1)$, and 0.528 is the slope of the Global Meteoric Water Line (GMWL)^8^. This definition is similar to the d-excess parameter calculated from δD and δ^18^O^9^. Evaporation causes low ^17^O-excess values in the residual water^8,10–12^. In the natural environment, ^17^O-excess decreases and δ^18^O increases systematically during evaporation under non-recharge conditions^13^. This results in typical evaporation curves in ^17^O-excess over δ′^18^O, where *h* is the primary control on curvature. Except for ambient water vapor δ^18^O_*V*_, all other variables have an effect inside the current analytical precision of ±8 per meg (1 sd) in ^17^O-excess over a large δ^18^O range. If δ^18^O_*V*_ remains reasonably well constrained, the triple O-isotope system may be used to quantify humidity and evaporative loss.

With the purpose to constrain the triple O-isotope systematics for the common case of evaporation and recharge, we here present new data from a series of groundwater-recharged ponds from the Salar de Llamara in the hyperarid Atacama Desert, Chile. Under the general absence of local rainfall, the ponds are hydrologically balanced by groundwater inflow from a distal source and evaporation, causing a steady increase in salinity from pond to pond along the flow path. Using field data and a complementary pan-evaporation experiment on-site, we identify the triple O-isotope system’s effective environmental variables and demonstrate the general predictability of ^17^O-excess with a simple steady state model for the hydrologic source-sink setting. We also demonstrate the general applicability of the triple O-isotope system in paleo-environmental studies.

## Study Area and Samples

The Salar de Llamara is a salt flat in the southern Pampa del Tamarugal, central Atacama Desert, Chile, between the Coastal Cordillera and the Andes. Open water is rare in this hyperarid environment. A groundwater-fed salt lake situated at W 69°37′, S 21°16′ comprises a number of small ponds along a linear, 250 m subsurface flow path, (Supplementary Fig. S1). The ponds are between 0.5 and 2 m deep. Groundwater originates from an aquifer draining a large alluvial fan on the western flank of the Andes^14^. Out of some 40 ponds in the salar, 11 were sampled. Groundwater – sampled from a well – is already salty (conductivity 5.8 mS/cm, TDS = 4.2 g/l). Conductivity increases along an evaporation gradient in the flow path from pond 11 with 24.2 mS/cm (TDS = 16.4 g/l) to pond 1 with 174.1 mS/cm, (TDS = 186 g/l) (Table 1). In addition, a pan evaporation experiment was conducted on site with three waters of different salt content over the period of three days. We used local tap water from Pica (0.8 mS/cm and TDS = 0.2 g/l), low-TDS water taken from pond 8 (37.2 mS/cm and TDS = 22.5 g/l), and high-TDS water from pond 1 (see above). Temperature and relative humidity was monitored on site. Rainwater from a rare event near Antofagasta (260 km south of the lake) during the 2015 El Niño complements the sample set. Supplementary Information (S1) contains additional details on climate and hydrological conditions, as well as on sampling and the experiment.

**Table 1:** Oxygen isotope data (SMOW-SLAP scaled) of evaporation experiment waters and natural samples.

		Sample	δ′^17^O	±(1 sd)	δ′^18^O	±(1 sd)	^17^O-excess	±(1 sd)	n	cond.	TDS
			(‰)	(‰)	(‰)	(‰)	(per meg)	(per meg)		(mS/cm)	(g/l)
		experiments									
tap water		exp L0	−6.682	0.165	−12.677	0.322	11	6	4	—	0.2
		exp L1	−4.446	0.116	−8.440	0.237	10	9	4	—	0.2
		exp L2	−3.492	0.189	−6.606	0.388	−3	16	4	—	0.3
		exp L3	−1.040	0.100	−1.957	0.201	−7	6	4	—	0.3
		exp L4	−0.832	0.270	−1.561	0.513	−7	5	7	—	0.4
		exp L5	1.455	0.203	2.807	0.380	−26	7	6	—	0.4
low-TDS water		exp LS0 (pond 8)	−2.011	0.658	−3.809	1.261	0	7	12	37.2	22.5
		exp LS1	−0.155	0.015	−0.282	0.024	−7	2	4	—	31.0
		exp LS2	0.182	0.045	0.364	0.097	−10	7	4	—	33.3
		exp LS3	1.910	0.049	3.686	0.107	−36	7	4	—	43.6
		exp LS4	1.928	0.147	3.735	0.282	−44	5	6	—	43.9
		exp LS5	4.260	0.202	8.195	0.385	−67	7	5	—	58.5
high-TDS water		exp HS0 (pond 1)	5.117	0.842	9.826	1.580	−72	6	10	174.1	186
		exp HS1	5.353	0.195	10.292	0.394	−81	14	4	—	213
		exp HS2	4.938	0.007	9.541	0.044	−100	16	4	—	249
		exp HS3	6.018	0.080	11.592	0.176	−102	12	4	—	292
		exp HS4	4.981	0.044	9.638	0.088	−108	2	4	—	286
		exp HS5	6.367	0.188	12.284	0.376	−118	13	7	—	392
	natural waters										
	group	pond									
		pond 8	−2.011	0.658	−3.809	1.261	0	7	12	37.2	22.5
		pond 11	−1.685	0.025	−3.205	0.030	7	10	4	24.2	16.4
	**I**	**average**	−**1.848**	**0.231**	−**3.507**	**0.427**	**4**	**5**			
	**II**	**pond 7**	−**0.579**	**0.084**	−**1.086**	**0.153**	−**5**	**3**	4	48.4	32.3
		pond 6	0.671	0.148	1.303	0.270	−17	6	4	63.1	47.7
		pond 9	1.082	0.005	2.087	0.020	−20	6	4	55.8	42.7
		pond 10	0.515	0.044	1.006	0.065	−16	10	4	58.5	38.8
	**III**	**average**	**0.756**	**0.293**	**1.465**	**0.558**	−**18**	**2**			
		pond 4	2.209	0.039	4.232	0.080	−26	3	4	90.5	65.7
		pond 5	1.716	0.020	3.307	0.013	−30	13	4	74.9	59.4
	**IV**	**average**	**1.962**	**0.348**	**3.769**	**0.654**	−**28**	**3**			
		pond 2	3.463	0.093	6.655	0.189	−51	10	6	158.8	141
		pond 3	3.655	0.004	7.022	0.004	−53	6	4	130.7	108
	**V**	**average**	**3.559**	**0.136**	**6.838**	**0.259**	−**52**	**1**			
	**VI**	**pond 1**	**5.117**	**0.842**	**9.826**	**1.580**	−**72**	**6**	10	174.1	186
		**average salar water**	**2.703**	**1.069**	**5.208**	**2.022**	−**48**	**10**			131
		site 12 (groundwater)	−3.328	0.155	−6.315	0.255	7	21	4	5.8	4.18
		rain	−2.840	0.059	−5.418	0.119	20	6	2	—	—

## O-Isotope Systematics During Evaporation

### Simple Evaporation

**Figure 1:**
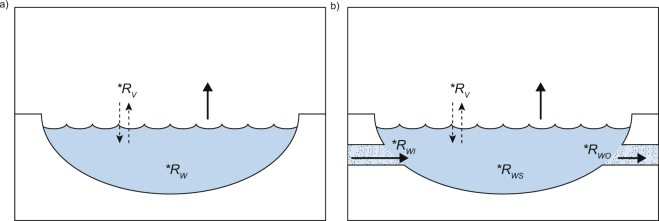
Conceptual models of natural water bodies in different hydrological settings. (**a**) Simple evaporation. (**b**) The hydrologic setting of the Salar de Llamara ponds with recharge and evaporation.

For simple (pan) evaporation (Fig. 1a), the isotopic composition (*R_*W*_) of a water body can be approximated by^7^:

4 $${}^{\ast }R_{W}={f}^{u}\cdot ({}^{\ast }R_{WI}-{}^{\ast }R_{SS})+{}^{\ast }R_{SS},$$

where *f* is the residual fraction, *R_*WI*_ is the isotopic composition of the initial water, and *R_*SS*_ is the predicted isotopic end value dictated by *h* and the isotopic composition of atmospheric vapor (*R_*V*_)^7^:

5 $${}^{\ast }{R}_{SS}=\frac{{}^{\ast }{\alpha }_{l-v{\rm{\_}}eq}\cdot h\cdot {}^{\ast }{R}_{V}\,}{1-{}^{\ast }{\alpha }_{l-v{\rm{\_}}evap}^{0}\cdot (1-h)},$$

The exponent *u* describes the fractionation factor as a function of relative humidity (*h*):

6 $$u=\frac{1-{}^{\ast }{\alpha }_{l-v{\rm{\_}}evap}^{0}\cdot (1-h)}{{}^{\ast }{\alpha }_{l-v{\rm{\_}}evap}^{0}\cdot (1-h)}$$

**Figure 2:**
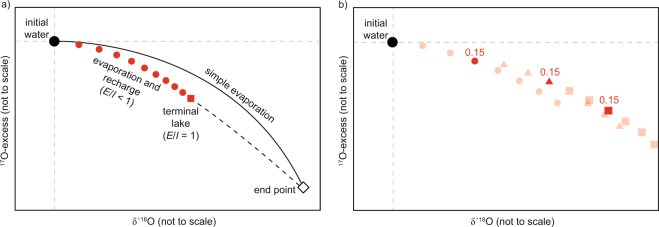
(**a**) Conceptual comparison of water isotopic composition for simple (pan) evaporation and recharge-balanced evaporation. In the simple evaporation case, evaporating water evolves along a trajectory (solid line) towards the isotopic end-point (white diamond). At the end-point, diffusion fractionation is balanced by equilibrium fractionation^7^. The value of the isotopic end-point depends on *R_*v*_ and *h* (equation (5)). Groundwater recharge (mixing) drives the water’s isotopic composition along a trajectory below the simple evaporation trend (red dots). Increasing *E*/*I* leads to higher δ′^18^O and lower ^17^O-excess up to a value of *E*/*I* = 1 where all inflow is balanced by evaporation (i.e. a stable, terminal lake, red square). The dashed line indicates situations where evaporation exceeds inflow and the water body shrinks. (**b**) Visualized pond-to-pond recharge model used in this study, shown for a hypothetical series of three ponds. Red symbols indicate different hydrologic steady states at variable *E*/*I* values for pond 1 (dots), pond 2 (triangles), and pond 3 (squares). The best fit for all but the last of the Salar de Llamara ponds was obtained for *E*/*I* = 0.15. Other *E*/*I* values are shown as shaded points and depict the respective evaporation trajectories. Modeling starts from initial groundwater. Pond 1 water assumes the respective isotopic composition at *E*/*I* = 0.15. Pond 2 will assume a composition for *E*/*I* = 0.15 on a new trajectory beginning at pond 1, and so on for subsequent ponds.

Here, *${\alpha }_{l-v{\rm{\_}}evap}^{0}\,$ is the effective fractionation factor for a hypothetical relative humidity of 0. In general, the effect of *R_*V*_ becomes larger with increasing *h*. If *h* > 0.5, equilibrium exchange between the water body and atmospheric vapor dominates *R_*W*_. If *h* < 0.5, evaporative flux dominates *R_*W*_, which causes steady isotopic enrichment in the water body^7,15^. In both cases an isotopic end-point, *R_*SS*_, is reached before the water body evaporates to dryness (Fig. 2a).

From equations (4)–(6) we calculated the expected isotopic composition trajectories of residual water for pan evaporation and compared calculations to measured results. We used equilibrium fractionation factors between ^18^*α*_*l*–*v_eq*_ = 1.01073 ± 0.00011 at 10 °C and ^18^*α*_*l*–*v_eq*_ = 1.00856 ± 0.00011 at 35 °C^16^. Values for ^17^*α*_*l*–*v_eq*_ follow from *θ*_*l*–*v_eq*_ = 0.529 ± 0.001^4^ (equation (1)). For diffusive fractionation we used ^17^*α*_*l*–*v_diff*_ = 1.0146 ± 0.0002 and ^18^*α*_*l–v_diff*_ = 1.0283 ± 0.0003 and *θ*_*l–v_diff*_ = 0.5185 ± 0.0002^5^. These authors found their values in good agreement with previous estimates. A very small temperature effect on *θ*_*l*–*v_diff*_ was tentatively suggested, but in the absence of rigorous experimental verification has not been considered in the present study.

In the absence of a direct measurement, the isotopic composition of atmospheric water vapor (*R_*V*_) was calculated from model rainfall composition in the *Online Isotopes in Precipitation Calculator* (*OIPC*) model^17^ to δ^18^O_*V*_ = −15.9‰ (see Supplementary Information S2.1). The *OIPC* model suggests a value for summer rain for Antofagasta of δ^18^O = −3.4 ± 0.8‰, which is in reasonable agreement with a rainwater sample collected there (δ^18^O = −5.4 ± 0.2‰). Data on ^17^O-excess_*V*_ of regional atmospheric vapor is presently not available. Values of ^17^O-excess_*V*_ in marine vapors range from −5 to +45 per meg^18^, with higher values found at low relative humidity. We assumed the GMWL value of ^17^O-excess_*V*_ = +33 per meg for local vapor based on the proximity of the Salar de Llamara to the Pacific coast (<50 km), low humidity along the coast, and the fact that the local meteoric water line of Northern Chile is identical to the GMWL in δD- δ^18^O^19,20^.

Two parametrizations are commonly required in equations (2), (5), and (6). These accommodate lower vapor pressure and slower evaporation of brines, and a reduced magnitude of *α_*l–v_diff*_ in a windy turbulent regime (Supplementary Information S2). These effects are addressed rigorously later in this study.

Evaporation at day and night were modelled separately to account for the large difference in temperature (10–35 °C) measured on site – which affects *α_*l–v_eq*_ – and *h* (0.2–0.8). The local average *h* = 0.43, weighted for the diurnal distribution of evaporation rates obtained from the evaporation experiment.

### Natural Evaporation with Recharge

In the ponds of the Salar de Llamara, hydrological balance is determined by inflow of groundwater (*I*), outflow (*O*) and evaporation (*E*). At steady state, *I* = *O* + *E* (Fig. 1b). Assuming well-mixed ponds, the isotopic composition is^7^:

7 $${}^{\ast }{R}_{WS}=\frac{{}^{\ast }{\alpha }_{l-v{\rm{\_}}evap}^{0}\cdot (1-h)\cdot {}^{\ast }{R}_{WI}+{}^{\ast }{\alpha }_{l-v{\rm{\_}}eq}\cdot h\cdot E/I\cdot {}^{\ast }{R}_{V}}{E/I+{}^{\ast }{\alpha }_{l-v{\rm{\_}}evap}^{0}\cdot (1-h)\cdot (1-E/I)}.$$

When *E*/*I* increases, *R_*WS*_ evolves towards the isotopic end-point – *R_*SS*_, see equation (5) – for evaporation at given *h* (Fig. 2a). Parametrizations for salinity and wind apply as in equations (5) and (6).

Evaporation trajectories can be modelled as a function of *E*/*I*. All other variables are known or were measured with the exception of *R_*V*_, whose annual average was estimated from the *OIPC* rain model to δ^18^O_*V*_ = −15.3‰^17^. The ponds of the Salar de Llamara may be modelled as a single terminal lake with a total salar average isotopic composition. The robustness of the model, however, is better verifiable for an evaporation series – here simplified by averaging ponds of similar composition (Table 1, groups I-VI). This also allows to account for the salinity effect on brine evaporation^21^. The terminal lake model, on the other hand, serves to demonstrate the principal applicability of ^17^O-excess measurements for paleo-*h* reconstruction.

## Methods

H_2_O was analyzed for O-isotopes on O_2_ by dual-inlet mass spectrometry on a *Thermo Scientific MAT 253*. O_2_ was extracted by fluorination^4^, with minor modifications^13^. In brief, 2.7 µl of water is injected into a CoF_3_ reactor at 370 °C. Liberated O_2_ is then cryogenically purified and transferred onto a 5 Å molecular sieve. The average long-term external reproducibility (1 sd) of our internal standard is ±0.120‰ for δ^17^O, ±0.250‰ for δ^18^O, and ±0.008‰ (8 per meg) for ^17^O-excess.

Oxygen isotope data in this study are SMOW-SLAP scaled^22^ and expressed against VSMOW-2 (Table 1). SLAP-2 measurement values against VSMOW-2 are −28.996‰ (δ^17^O) and −54.172‰ (δ^18^O). To exclude systematic isotope effects of salt on water fluorination, artificial samples of the same water with up to 120‰ salt content, respectively, were analyzed and found to be equally reproducible (Supplementary Table S1). Hydrogen isotopes in water were measured by continuous flow analysis of H_2_ liberated by carbon reduction in a *HEKAtech HT 1700 oxygen analyser* (Supplementary Table S2).

Major dissolved ions (Na^+^, K^+^, Mg^2+^, Ca^2+^, Cl^−^, and SO_4_^2−^ as total S) were analyzed by ICP-OES. Dissolved inorganic carbon was analyzed by titration. The concentration of HCO_3_^−^ and CO_3_^2−^, pH, and activities of all dissolved major ions were approximated by specific ion interaction modeling using the PHREEQC software package^23,24^. Supplementary Table S3 summarizes all chemical data along with TDS – calculated as sum of all measured ions.

### Data availability statement

All data generated or analyzed during this study are included in this published article (and its Supplementary Information files).

## Results

### Isotope Data

**Figure 3:**
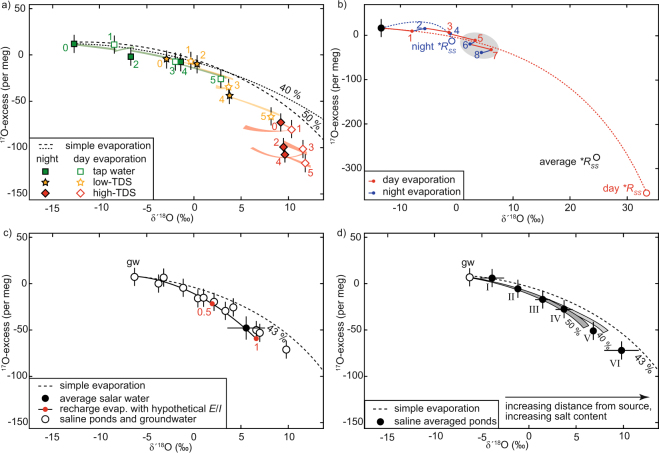
(**a**) Oxygen isotope data from the pan evaporation experiment. The dashed lines are model curves for simple (pan) evaporation into a natural atmosphere at average relative humidity 0.4 and 0.5, respectively. The colored trajectories are model curves from sample to sample, taking into account the diurnal *h*-cycle (day: *h* = 0.35, night: *h* = 0.80) and the salinity effect. Numbers indicate the sample’s position in the respective sampling sequence of the three evaporation experiments. Odd numbers represent evaporation at day, even number at night. Vapor composition (δ^18^O_*V*_ = −15.9‰) and wind turbulence (*n* = 0.5) are kept constant. (**b**) Model of a pan evaporation trajectory as in a) but extrapolated over four days for the given evaporation rate, the diurnal *h*-cycle, and its effect on shifting isotopic day and night end-points (*R_*SS*_). Circles represent respective isotopic end-points. Numbers indicate the evaporation sequence. (**c**) Isotopic composition of individual ponds and of average total salar water (asw), modelled trajectories at local average *h* = 0.43 for simple evaporation and for evaporation with recharge including hypothetical *E*/*I* of 0.5 and 1 (red dots). Error bars represent 1 sd of the average for δ′^18^O, and the external analytical reproducibility for ^17^O-excess. (**d**) Modelled evaporative succession of groundwater (gw) and ponds (averaged where composition was similar, roman numerals, see also Table 1). Grey envelopes illustrate modelled series of steady states for *E*/*I* = 0.15 at given boundary conditions and two different *h* (0.4 and 0.5) with an uncertainty of ±0.5‰ in the estimate of ambient atmospheric δ^18^O_*V*_.

Evaporation results in a systematic increase in δ′^18^O and a decrease in ^17^O-excess (Fig. 3a,c). In the experiment, evaporation rates and isotope fractionation decrease measurably with salinity. For the region’s tap water (TDS = 0.2 g/l), evaporation loss was 58% and Δδ′^18^O_end-start_ = 15.5‰. For the low-TDS water (TDS = 22.5 g/l), loss was 56% and Δδ′^18^O_end-start_ = 12.0‰. For the high-TDS water (TDS = 186 g/l), loss was 50% and Δδ′^18^O_end-start_ = 2.5‰. The three experiment samples appear to evolve along a common evaporation trend, but fall below the evaporation curve expected for simple evaporation at the site’s given average *h* = 0.43 (Fig. 3a). A reversal of trend in δ′^18^O occurs during evaporation at night in the more evaporated low-TDS samples (from 3 to 4 in Fig. 3a) and the high-TDS samples (from 1 to 2, and 3 to 4 in Fig. 3a).

Measured δ′^18^O = −6.3‰ for local groundwater is consistent with data reported elsewhere from the Atacama region, (−5.5 to −6.5‰)^25,26^. The ponds increase in δ′^18^O (Δδ′^18^O = 16.1‰) and decrease in ^17^O-excess (Δ^17^O-excess = −79 per meg) with increasing salinity (Fig. 3c). All ponds plot close to the recharge evaporation curve modelled with *h* = 0.43 for a single terminal lake of total salar average composition (δ′^18^O = 5.2‰ and ^17^O-excess = −48 per meg, weighted according to pond surface area). The overall experiment appears to progress along a trend unexpectedly similar to the model curve for the terminal lake despite the fact that evaporation of the latter also depend on the ratio of *E*/*I* – *cf*. equations (4) and (7). However, groundwater recharge in the ponds and the diurnal trend reversal in the experiment– i.e mixing – may coincidentally have comparable effects on evaporation trajectories.

### ICP-OES Data and Alkalinity

The brines classify as Na-Cl-SO_4_^27,28^. TDS increases from groundwater to the highly evaporated pond 1 by more than an order of magnitude (Supplementary Table S3). All ions except Ca^2+^ show a continuous enrichment with increasing evaporation. Modelled ion activities of Ca^2+^ and dissolved inorganic carbon species suggest saturation of groundwater and all ponds with respect to Calcite/Aragonite. As Ca^2+^/Cl^−^, Mg^2+^/Cl^−^ and HCO_3_^−^/Cl^−^ molar ratios also decrease, continuous carbonate precipitation is likely. The modelled SO_4_^2−^ and Ca^2+^ ion activity product remains close to gypsum saturation in all ponds. Gypsum precipitation is visible in all ponds and generally associated with algae mats. An abundance of selenite gypsum crystals in pond 1 and to a lesser extent in ponds 2 and 3 suggest at least partial inorganic gypsum precipitation^29^.

An increase in Na^+^/Cl^−^ between groundwater and the first ponds along with a decrease in all other ion/Cl^−^ ratios suggests subsurface dissolution of a sodium salt on groundwater ascent. Circumstantial evidence points to sodium sulfate. Highly hygroscopic sodium sulfate precipitated from the evaporated high-TDS pan experiment samples. In a common Na-Cl-SO_4_ type salar with abundant gypsum precipitation, secondary subsurface formation of double and triple sulfate salts like glauberite (with Na^+^ and Ca^2+^), polyhalite (with K^+^, Mg^2+^ and Ca^2+^) and bloedite (with Na^+^ and Mg^2+^) may also occur^27^. K^+^ and Cl^−^ are the most conservatively behaving ions and were used to estimate *E*/*I* for averaged ponds of similar composition (Supplementary Table S4). However, these estimates are rather variable and most likely provide only broad constraints. The *E*/*I *estimate for ponds of the least saline group I is unrealistically high (0.83). At *E*/*I* close to unity – the state of a terminal lake – inflow would have to be exceptionally high in order to sustain the equally large but an order of magnitude more saline pond 1 (group VI) downstream at the end of the flow path. It is rather likely, that subsurface dissolution during groundwater ascent affects K^+^ and Cl^−^ as well. The high variability of *E*/*I* between individual pond-groups is not reproduced when estimated from the isotope data (see below).

## Discussion

To verify the robustness of the terminal lake model, we also modelled pond-to-pond evaporation trajectories for six averaged groups of ponds with similar composition (Table 1). The averaging was done to avoid complex model outcomes between ponds of small compositional difference. The two parametrized effects – sluggish evaporation of brines and wind turbulence – are briefly outlined here and are explained in detail in the Supplementary Information (S2). First, lower vapor pressure of brines slows their evaporation^21^. This may be parametrized by replacing actual *h* in equations (5)–(7) with a higher effective humidity *h*_*eff*_ calculated from brine density. Second, the contribution of kinetic fractionation in *${\alpha }_{l-v{\rm{\_}}evap}^{0}$ – equation (2) – is smaller in a high wind regime due to turbulence at the water-air boundary^30^. This effect may be parametrized by introducing an exponent *n* to the fractionation factor for diffusion (*${\alpha }_{l-v{\rm{\_}}diff}^{n}$). Local wind data was unavailable, therefore the empirical estimate of *n* = 0.5 for rough wind regimes was used^31,32^.

Calculating *h*_*eff*_ from water sample density for the high-TDS water experiment samples yields *h*_*eff*_ = 0.9 at night, instead of measured *h* = 0.78 and *h*_*eff*_ = 0.37 during the day instead of *h* = 0.32 (Supplementary Table S5). This is in principle a sufficient difference as to affect model evaporation curves. However, the apparent reversal in δ′^18^O of progressively evaporated high-TDS samples during the night in comparison to the previous daytime samples (Fig. 3a) reveals that an additional physical process related to diurnal variability in *h*_*eff*_ is responsible for the unexpectedly high curvature of the evaporation trajectory. The reversal is not likely the result of changing isotopic composition of atmospheric vapor (*R_*V*_). Instead, the observed pattern is demonstrably the result of day and night changes of the *R_*SS*_ (equation (5)) - the isotopic end-point towards which evaporation progresses. The diurnal temperature cycle and its effect on *h* (Fig. 3b) results in a diurnal cycle of *R_*SS*_. This effect may be modelled with reasonable accuracy by using two averaged end-members for day and night *h*. Once the water body’s actual isotopic composition during the day has moved to the right of the night-*R_*SS*_ in the diagram – as in the high-TDS experiment – the evaporation trend reverses at night, leading to an overall zig-zag pattern as evaporation progresses over several days toward the diurnal average *R_*SS*_ value.

The apparent common simple evaporation trajectory in the experiment of tap water with respect to low-TDS water and high-TDS water taken from the salar, might be explained by evaporation under common conditions, assuming that regional tap water and Salar de Llamara groundwater principally originate from isotopically similar sources – i.e. the western flank of the high Andes. The small difference between the isotopic composition of actual Salar de Llamara groundwater (δ′^18^O = −6.3‰, ^17^O-excess = 7 per meg) and tap water (δ′^18^O = −12.7‰, ^17^O-excess = 11 per meg) could be the result of some evaporation through the soil along the aquifer’s flow path^20^ (Fig. 3a). However, the general flatness of curves in ^17^O-excess over δ′^18^O at early stages of evaporation makes it difficult to discriminate evaporation clearly from a simple difference in isotopic source composition. D/H data suggest that the latter is the more likely explanation (see below).

The evaporation trend between pond-groups of similar composition (*R_*WS*_) was modelled using equation (7) with *E*/*I* as the free variable for *h* = 0.4 and 0.5 and including the above parametrizations (Fig. 3d). Here, local groundwater or the preceding pond-group determine the recharge isotopic composition of the succeeding pond-group *R_*WI*_. All other boundary conditions are summarized in Supplementary Table S6. Pond-to-pond modeling produces a constant *E*/*I* = 0.15 for all pond-groups except for the most saline, where *E*/*I* = 0.3. This is lower and less scattered than the average independent estimate based on Cl^−^ and K^+^, where *E*/*I* = 0.45 and significant scatter is present between pond-groups (Supplementary Table S4). However, subsurface dissolution of older salt may compromise this approach (see above). Modeling *E*/*I* values from isotope data, on the other hand, depends on correctly assuming the inflow composition. Taking the value from a preceding pond may not be correct if there is subsurface flow^29^, for which there is evidence from mass balance consideration and from observation. The surface area of the final and most saline pond (pond 1/group VI) is 52% of the total salar water surface. The much smaller preceding ponds are only a fraction in size and cannot sustain that much outflow. Ubiquitous salt efflorescence between ponds indicates evaporation through the salt surface by capillary suction. Rates are most likely different for open water and capillary evaporation. Therefore, less evaporated water of a different composition may flow below the salar surface. This would lead to an overestimate of δ′^18^O and underestimate of ^17^O-excess for inflow-*R_*WI*_ and to false estimates of *E*/*I* and curvature of the evaporation trajectory. The total salar average of *E*/*I* = 0.9 (Fig. 3c) and visible salt efflorescence suggest some subsurface flow leaking beyond the most saline pond.

The observation that the evaporation trajectory of the pan experiment is so similar to that of the successive ponds could suggest that the effect of a diurnal cycle of the isotopic end-point also applies to the recharged ponds. Some scatter between a few repeat samples taken from pond 1 and pond 8 could hint at an under-sampled diurnal stratification-mixing cycle to that effect. However, the daily mixing induced by strong afternoon winds and waves on the ponds’ surface, the ponds’ large volumes, and – above all – continuous recharge do not support a large diurnal isotopic cycle of the magnitude found in the small-volume pan experiment. Instead, the similarity of trajectories results from the coincidence that mixing with groundwater (δ′^18^O = −6.3‰, ^17^O-excess = 7 per meg) and night-time evaporation towards the respective isotopic end-point (δ′^18^O = −0.61‰, ^17^O-excess = −10 per meg) in the pan experiment pull the actual isotopic composition of the respective water bodies back in almost the same direction.

### Application

**Figure 4:**
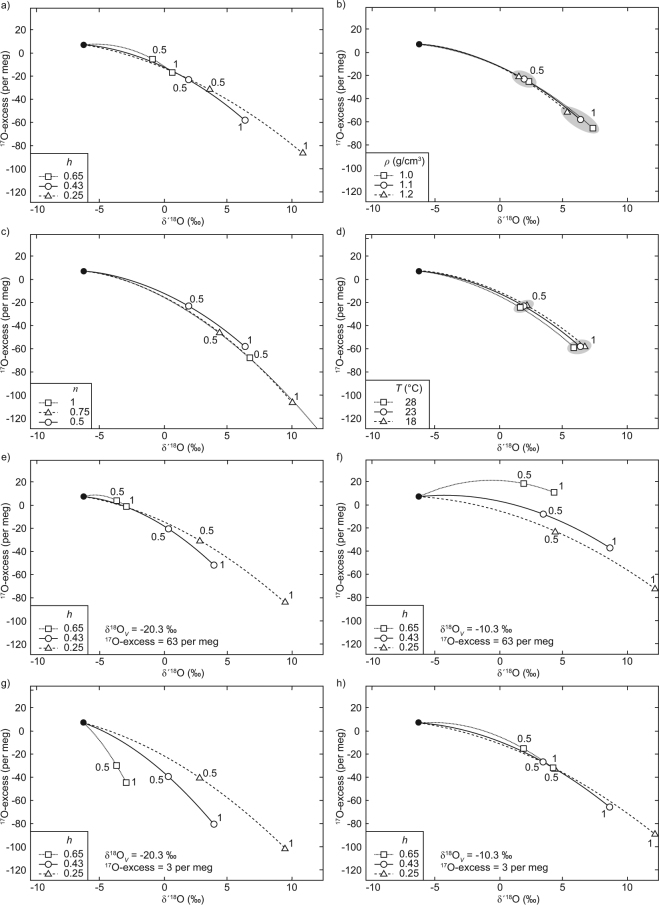
Model sensitivity in ^17^O-excess over δ′^18^O for different variables and parameters during evaporation and recharge for initial water with Salar de Llamara groundwater composition (black dot). Open symbols represent steady states for *E*/*I* of 0.5 and 1 at given *h*, respectively. Normal boundary conditions (solid line) are *ρ* = 1.1 g/cm^3^, *n* = 0.5, δ^18^O_*V*_ = −15.3‰, ^17^O-excess_*V*_ = 33 per meg, *h* = 0.43, *T* = 23 °C. **(a**) Evaporation at variable relative humidity (*h*). (**b**) Evaporation at variable density (*ρ*) – proportional to salt content – from which *h*_*eff*_ is calculated. (**c**) Evaporation at variable wind turbulence, ${}^{\ast }\alpha _{l-v\_diff}^{n}$. (**d**) Evaporation at variable water temperature (*T*). (**e**–**h**) Evaporation at variable δ^18^O_*V*_ (−20.3 to −10.3‰) and ^17^O-excess (3 to 63 per meg).

Triple-O isotopes may be useful for quantitative reconstruction of paleo-*h*, e.g. from structurally bonded water in sedimentary gypsum^29,33,34^. General applicability of this tool depends not so much on analytical uncertainty, but rather on how well evaporation trajectories of different *h* can be resolved in ^17^O-excess over δ′^18^O (Fig. 4a). If the given boundary conditions of the Salar de Llamara are assumed constant, a decrease in *h* will result in shallower and longer evaporation curves. Other boundary conditions, particularly with respect to vapor composition (*R_*V*_), may result in differently shaped evaporation trajectories (see below). For a through-flow lake, it is generally difficult to discriminate changes in *h* from changes in *E*/*I*. For a terminal lake – where *E*/*I* can be assumed within the range of 0.9–1.0 – variability in local boundary conditions other than *h* and *R_*V*_ either leads to negligible uncertainty in estimated paleo-*h*, or is detectable because of an isotopic state in ^17^O-excess over δ′^18^O that may not reasonably be achieved by a change in paleo-*h*.

A doubling of the salinity effect by a change in brine density from *ρ* = 1.1 g/cm^3^ (TDS ~ 150 g/l) to *ρ* = 1.2 g/cm^3^ (TDS ~300 g/l) is equal to only a small change in *h*_*eff*_ from 0.47 to 0.52 – unresolvable at the site’s given boundary conditions (*cf*. Fig. 4a,b). Variable air turbulence generally has a small effect on the trajectory’s curvature, but a considerable effect on the length of the curve (*cf*. Fig. 4c,d). A change to a calmer wind regime – for example an exponent *n* = 0.75 instead of *n* = 0.5 – would move the isotopic state of a terminal lake far beyond the point where *E*/*I* = 1 on a curve that cannot be explained by a change in *h*. A terminal lake under a lower *h* by 0.2 is ~2‰ in δ′^18^O and ~20 per meg in ^17^O-excess apart from one with unchanged *h* but with a calmer wind regime and *n* = 0.75. Changes in temperature result in no detectable change in ^17^O-excess but in δ′^18^O (Fig. 4d). If analytical uncertainty of 5 per meg in ^17^O-excess is considered in addition to a 5 °C temperature change (~ 0.5‰ change in δ′^18^O), the uncertainty in paleo-*h* would be about 0.05.

A potentially large effect may arise when vapor composition (*R_*V*_) changes relative to initial water composition (*R_*WI*_). Evaporation trajectories are well distinguishable for different *h* and associated isotopic states for terminal lakes if vapor and water are isotopically relatively different (Fig. 4f,g). *R_*V*_ and *R_*WI*_ may vary independently from each other in climates with a pronounced seasonal distribution of rain and drought or seasonally changing wind direction and moisture source^35^. Evaporation trajectories for different *h* merge, when *R_*V*_, *R_*WI*_, and *R_*WS*_ fall close to a common line in ^17^O-excess over δ′^18^O (Fig. 4e,h). This may be the case even if the differences in δ′^18^O and ^17^O-excess are large (Fig. 4e) Additional uncertainty in the reconstruction may come from variability in the isotopic composition of inflow (*R_*WI*_). This issue may be addressed by complementary δD analysis.

**Figure 5:**
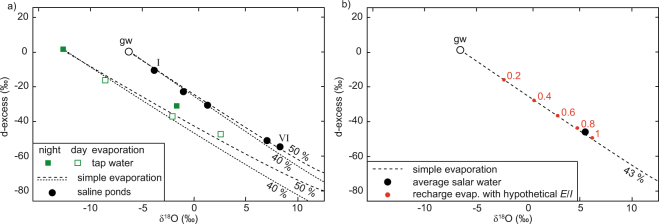
(**a**) δ^18^O and d-excess data of selected waters from pan evaporation experiments and for Salar de Llamara ponds with modelled simple evaporation trajectories (no recharge). (**b**) Modelled evaporation trajectory for recharge with individual *E*/*I* and the total Salar de Llamara average. Error bars (1 sd) are smaller than symbol size.

The d-excess and ^17^O-excess parameters have some notable differences and complementary advantages. The lower fractionation dependency on temperature for *θ* calculated from ^17^O/^16^O and ^18^O/^16^O^4,16,36^ is only a minor advantage because (temperature sensitive) δ′^18^O is always required as well to obtain unique hydrological information from ^17^O-excess (*cf*. Fig. 4d and Supplementary Fig. S2d). Also, relative analytical uncertainty in ^17^O-excess is comparatively large. The d-excess parameter may suffer from a potentially more complex salt effect. The relationship between δD and δ^18^O is sensitive to changes in salt composition of the brine as well as its salinity (Supplementary Information S2.2.1). Trajectories in d-excess over δ^18^O system are also more sensitive to variable air turbulence (*cf*. Fig. 4c and Supplementary Fig. S2c). Both systems are sensitive to large variations in *R_*V*_ relative to *R_*WI*_ (*cf*. Fig. 4e to h and Supplementary Fig. S2e to 2h). In d-excess over δ^18^O, a change in inflow composition (*R_*WI*_) is better distinguishable from an early stage evaporative change in *R_*WI*_, because of insensitivity in ^17^O-excess at the flat initial evaporation trajectory (*cf*. Figs 3a and 5a). The d-excess parameter does not support the earlier (see above) discussed possibility that Salar de Llamara groundwater and local tap water evaporated on a common trajectory during the on-site pan experiment. For the boundary conditions at the Salar de Llamara, the d-excess parameter does not discriminate well between simple and recharged evaporation (Fig. 5b)

## Conclusions and Outlook

This study demonstrates that relative humidity (*h*), evaporation over inflow (*E*/*I*), the isotopic composition of vapor (*R_*V*_), and of inflowing water (*R_*WI*_) determine ^17^O-excess of evaporated water in a recharged lacustrine environment. Wind turbulence is well accounted for by using *${\alpha }_{l-v{\rm{\_}}diff}^{n}$ with *n* = 0.5. Uncertainty in wind turbulence has a small effect on trajectory curvature but some effect on a lake’s position on the curve, i.e. its *E*/*I*. The salt effect due to sluggish evaporation of brines is a comparatively small source of uncertainty. The applicability of the ^17^O-excess parameter to resolve changes in paleo-*h* from measurements of authigenic minerals preserving past water O-isotope ratios, such as carbonates^37^ or gypsum hydration water^29,33,34^, depends strongly on the composition of *R_*V*_ relative to that of *R_*WI*_. If that compositional difference is large enough to achieve the required resolution in *h*, paleo-*h* may be reconstructed for terminal lakes from single measurements. Without prior knowledge of *E*/*I* – e.g. in through-flow lake – the reconstruction of paleo-*h* may still be possible. Variability in *E*/*I* at constant *h* over time should result in a spread of down-core measurements plotting on a single evaporation trajectory largely determined by ambient *h*. Large enough changes in *h* should produce a spread of measurements falling on multiple trajectories. In both cases, complementary d-excess data are useful to constrain variability in boundary conditions and may allow better separation of changes in *h* from changes in inflowing δ^18^O_*WI*_.

Another field of application is hydrologic balancing of present day lakes and aquifers. If *h* and the absolute evaporation rate can be monitored independently, the monitoring of triple O-isotopes in lake water or an aquifer should allow a reasonably precise absolute estimate of the inflow rate and assessment of its variability over time. This may principally also be achieved with d-excess data. However, with complementary ^17^O-excess data, hydrologic balancing of an aquifer may be possible even when there is great spatial heterogeneity in wind turbulence and in *h*.

## Electronic supplementary material

Supplementary Information
