# Author Correction: The evolution of ^17^O-excess in surface water of the arid environment during recharge and evaporation

**DOI:** 10.1038/s41598-019-43264-w

**Published:** 2019-05-09

**Authors:** J. Surma, S. Assonov, D. Herwartz, C. Voigt, M. Staubwasser

**Affiliations:** 1 Institute of Geology and Mineralogy, University of Cologne, Cologne, Germany; 2 Present Address: International Atomic Energy Agency, Vienna, Austria

Correction to: *Scientific Reports* 10.1038/s41598-018-23151-6, published online 21 March 2018

This Article contains an error in Reference 26 which was incorrectly given as:

Scheihing, K., Moya, C., Struck, U., Lictevout, E. & Tröger, U. Reassessing Hydrological Processes That Control Stable Isotope Tracers in Groundwater of the Atacama Desert (Northern Chile). *Hydrology* **5**, 3, 10.3390/hydrology5010012 (2017).

The correct reference is listed below:

Scheihing, K., Moya, C., Struck, U., Lictevout, E. & Tröger, U. Reassessing Hydrological Processes That Control Stable Isotope Tracers in Groundwater of the Atacama Desert (Northern Chile). *Hydrology* **5**, 3, 10.3390/hydrology5010003 (2017).

