# Supplementary material for: The evolution of 17O-excess in surface water of the arid environment during recharge and evaporation
Source: Sci Rep. 2018 Mar 21;8:4972. doi: 10.1038/s41598-018-23151-6 (PMC5862851; doi:10.1038/s41598-018-23151-6)
Supplement: Supplementary file 1 — Supplementary Information [file 41598_2018_23151_MOESM1_ESM.pdf]

Supplementary Information

The evolution of ^{17}O-excess in surface water of the arid environment during recharge and evaporation

Surma, J.[1*], Assonov, S.[1,2], Herwartz, D.[1], Voigt, C.[1], and Staubwasser, M.[1]

[1]Institute of Geology and Mineralogy, University of Cologne, Cologne, Germany

[2]formerly at Institute of Geology and Mineralogy, University of Cologne, Cologne, Germany; presently at International Atomic Energy Agency, Vienna, Austria

*corresponding author: jakub.surma@uni-koeln.de

S1 Study Area and Samples

The Salar de Llamara is a salt flat in the hyperarid Atacama Desert of Chile. At W 69°37', S 21°16' there is a salt lake recharged only by groundwater. Local rainfall is absent. The nearest weather station to the Salar de Llamara – at Pozo Almonte, 110 km north of the lake site – shows an average annual precipitation < 1 mm/yr. On average, easterly winds prevail with a speed of 5 – 15 m/s, but there is a thermally induced reversing diurnal pattern. Winds are strongest in the afternoon and blow with enough force to induce waves on the ponds and thus some extent of vertical mixing. Average temperature (*T*) is 18 to 25 °C from October to March and 14 to 19 °C from April to September. Relative humidity is low and may fall below 20 %. During the field campaign (March, 3rd to 7th) *T* and *h* were monitored on-site and showed a large intraday variation from 35 °C and 20 % *h* at the peak of the day, to 15 °C and 80 % during the night.

The ponds in the Salar de Llamara are shallow and between 0.5 and 1 m deep with the exception of pond 11, a sinkhole of about 2 m depth. Environmental protection measures have been enacted to counter a long-term decline in water level of the salar's remnant lake. A pumping station with three injection pipes branching off from a single supply-pipeline was constructed in late 2013 to refill the aquifer and prevent the lake from drying up. However, pumping had not yet been operational at the time of the sampling campaign in March 2014. Instead, groundwater water of low salinity was flowing through one of the injector pipes, rising under pressure from the sub-surface aquifer.

S1.1 Natural Water Samples

During the campaign, we sampled groundwater and 11 ponds in the remnant lake system (Fig. S1). Conductivity was measured on site, showing a range from 24.2 mS/cm to 174.1 mS/cm. Total

dissolved solids (TDS, calculated from major element analysis) range from 16.4 g/l (pond 11) to 186 g/l (pond 1). Water from the aquifer was sampled from a pressure valve on the injector pipe with flowing water (sample 12, conductivity = 5.8 mS/cm and TDS = 4.2 g/l. In addition, we sampled a rare rainfall event near Antofagasta (260 km south of the lake) during the 2015 El Niño.

S1.2 Evaporation Experiment

A pan evaporation experiment was set up between the salar's ponds to investigate isotopic effects during evaporation without recharge for comparison. Three different waters - 1.2 l of local tap water (Pica, 90 km northeast of the Salar de Llamara, TDS = 0.2 g/l), water from pond 8 (TDS = 22.5 g/l) and water from pond 1 (TDS = 186 g/l) - were filled into stainless steel pans (20 cm diameter). The experiment was conducted over three days and sampling was performed around 10:00 am and around 5:00 pm, starting in the evening of the first day after setting up the experiment in the morning. TDS of water from pond 8 increased to 59 g/l, that of pond 1 increased to 392 g/l over the course of the experiment. Evaporation of these saline waters was accompanied by precipitation of gypsum and – in pond 1 water – of another soluble mineral, presumably mirabilite ($Na_2SO_4 \cdot 10H_2O$) as indicated by water chemistry and previously described[1]. The average fraction of water lost during the day was $f_d = 0.2 \pm 0.05$ and during the night was $f_n = 0.07 \pm 0.02$ (see Table S5 for detailed information). Extrapolated evaporation rates to the year are 3,000 mm/yr (saline water) to 3,500 mm/yr (tap water). These are in good agreement with reported annual average rates of 3,500 to 4,000 mm/yr[2]. Total evaporation can be estimated using the open water surfaces (approx. 5,600 m^2) and the average annual evaporation (3,500 mm m/yr). This results in a total evaporation loss of approx. 18,000 m^3/yr (50 m^3/d). However, this estimate does not account for evaporation through the ground, which is clearly indicated by salt efflorescence between the ponds and the surroundings of the lake system.

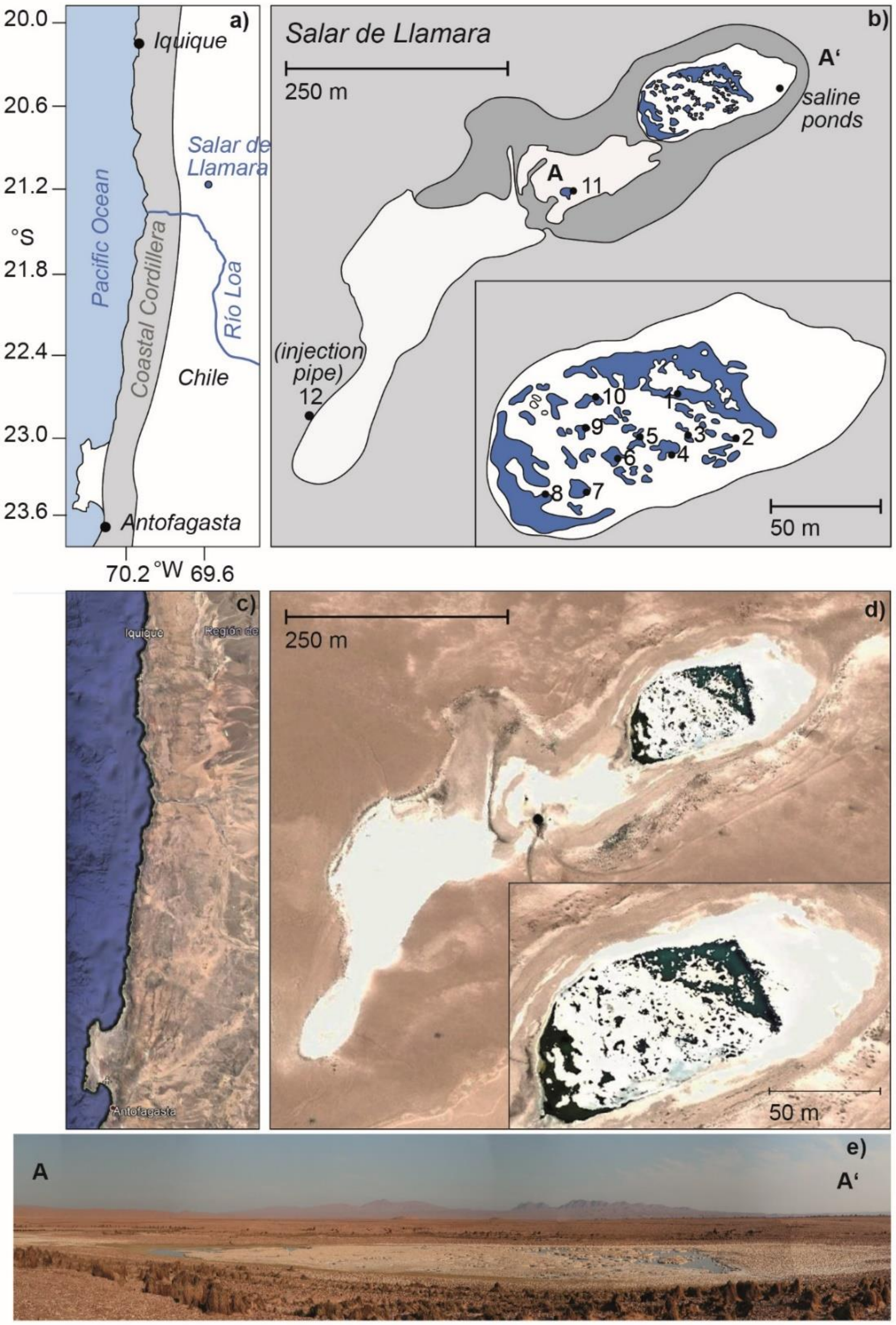

Figure S1: a) Location of the Salar de Llamara within the Atacama Desert of Chile, **b)** local map, **c)** and **d)** aerial photos, **e)** Photograph of the salt lake. Photographs in **c)** and **d)** are taken from Google Earth Pro (https://www.google.com/earth/, v. 7.3.0), DigitalGlobe. Maps in **a)** and **b)** are modified from **c)** and **d)**.

S2 Parameterization

One indirectly estimated variable and two sensitive parameters affecting equations (5) – (7) are outlined in the following: These are the isotopic composition of atmospheric vapor ($^{*}R_{V}$), salinity effects on vapor pressure, i.e. the effective humidity (h_{eff}), and a wind turbulence correction (n).

S2.1 Isotopic Composition of Atmospheric Vapor

$^{*}R_{V}$ was not measured but approximated from the meteoric water composition. The empirical *Online Isotopes in Precipitation Calculator* (*OIPC*) model[3] suggests meteoric water $\delta^{18}O$ = -6.7 ± 0.5 ‰ in March. Measurements of rain collected in March 2015 in the area of Antofagasta show that $\delta^{18}O$ predicted by *OIPC* (-3.4 ± 0.8 ‰) is relatively close to our measured value of -5.40 ± 0.24 ‰. This suggests that the *OIPC* data of this region are a reasonably accurate basis for estimations of $\delta^{18}O_{V}$. This is done using $^{18}\alpha_{l-v\_eq}$ for the mean air temperature $T \approx 23$°C (March) and yields $\delta^{18}O_{V} \approx$ -15.9 ‰. For the annual mean, $\delta^{18}O$ = -5.5 ‰ and $T \approx 19$ °C, this yields $\delta^{18}O_{V}$ ≈ -15.3 ‰. We assume that ^{17}O-excess of atmospheric vapor is ≈ 33 per meg. The rationale for this assumption is outlined in the main text (see Discussion).

S2.2 Salinity

Two aspects should be considered for calculating isotopic effects during evaporation of brines from equations (5) – (7): The ‘classic’ salt effect influences isotope activities[4–6] and the humidity effect describes the decrease of vapor pressure above the fluid with increasing salt content[7].

S2.2.1 The “Classic” Salt Effect

The ’classic’ salt effect (Γ) describes the fact that $^{18}O/^{16}O$ and D/H activity ratios diverge from the corresponding concentration ratios ($R_{activity} \neq R_{composition}$). Γ quantifies the isotopic fractionation between free water ($^{*}\alpha_{v-l_{pure\,water}}$) and water in ionic hydration shells ($^{*}\alpha_{v-l\_solution}$)[4–6,8]. Since evaporation in natural systems depends on activity, all $^{*}\alpha_{v-l\_eq}$ ratios need to be corrected for the classic salt effect using the relationship: $\Gamma = {}^{*}\alpha_{v-l\_solution} / {}^{*}\alpha_{v-l\_pure\,water}$. $\Gamma_{^{18}O/^{16}O}$ is mainly controlled by cations in the solution[8] while $\Gamma_{D/H}$ is controlled by cations and anions[9,10]. Thus, d-excess is sensitive to the main ion composition of the water. The Na-Cl-SO_4 brines in the Salar de Llamara are dominated by NaCl and reach a 2.5 molal concentration in the saltiest pond. There is some controversy to the exact magnitude of the salt effect by NaCl. Some experimental studies have shown that NaCl has little effect on the isotopic activity of oxygen[11,12], while others report a significant effect[13]. This chemical salt effect is small, however, for the single element system of triple oxygen isotopes. As such, we assume $^{*}R_{activity} = {}^{*}R_{composition}$, and neglect the ‘classic’ salt effect in our model calculations.

S2.2.2 Effective Humidity

In addition to a chemical salt effect, there is a physical effect. Dissolved salt raises water viscosity and lowers the vapor pressure. Slower evaporation at lower vapor pressure is parametrized by performing calculations with equations (2) and (5) – (7) with a higher humidity than actual, the effective relative humidity h_{eff}. There are two methods for calculation of h_{eff} [7,14]. In the first approach, $h_{eff} = h \cdot P_0/P_S$ where P_0 is the vapor pressure of pure water and P_S is the vapor

pressure of the saline water[7]. The latter may be approximated from salinity with $P_S = P_0 \cdot (1 - 0.00407s - 0.000187s^2)$ at 20 °C[14]. In a second approach h_{eff} is estimated using the measured density of the brines and Raoult's law[14]:

$$h_{eff} = h\frac{\rho}{\rho_W}$$

Where ρ is the density of the brine and ρ_W is the density of pure water (taken as 0.9982 g/cm^3, density of water at 20 °C). For the brines analyzed in this study, both methods agree within 1.3 % for salinities up to 200 g/l. At very high salinity, Raoult's law yields systematically lower h_{eff} (by 3 % at 260 g/l). Both methods appear sufficiently accurate for the purpose of the present study with brine concentrations less than 190 g/l of dissolved solids. We chose to use Raoult's law to derive h_{eff} for use in the respective evaporation equations.

S2.2.3 Wind Effect and Turbulence

Wind turbulence influences the isotopic fractionation process by disturbing the diffusive layer above the water surface and by lowering of the effective fractionation, (${}^{*}\alpha_{l-v\_evap}$) [15–17]. The correction for turbulence is done by introducing a correction exponent *n* (${}^{*}\alpha_{l-v\_diff}^{n}$). The exponent *n* is determined empirically and typically ranges from 0.5 for turbulent air[18–20] to 1.0 for complete calm. Modeled isotope evaporation trajectories are a function of the fraction *f* of residual water (equation 4). From pan evaporation experiments the loss of water (*1-f*) for each experimental sample was measured. Isotope data best fit the modeled evaporation trajectory for *n* = 0.5. The use of the value for high turbulence seems justified at prevailing local wind speed between 5 and 15 m/s. Previous studies suggested the use of a universal ${}^{18}\alpha_{l-v\_diff}$ = 1.005[16] or ${}^{18}\alpha_{l-v\_diff}$ = 1.008[17]

for this range of wind speeds. However, these fractionation factors were determined from oceanic vapor measurements at considerably higher local values for *h* and ^{17}O-excess.

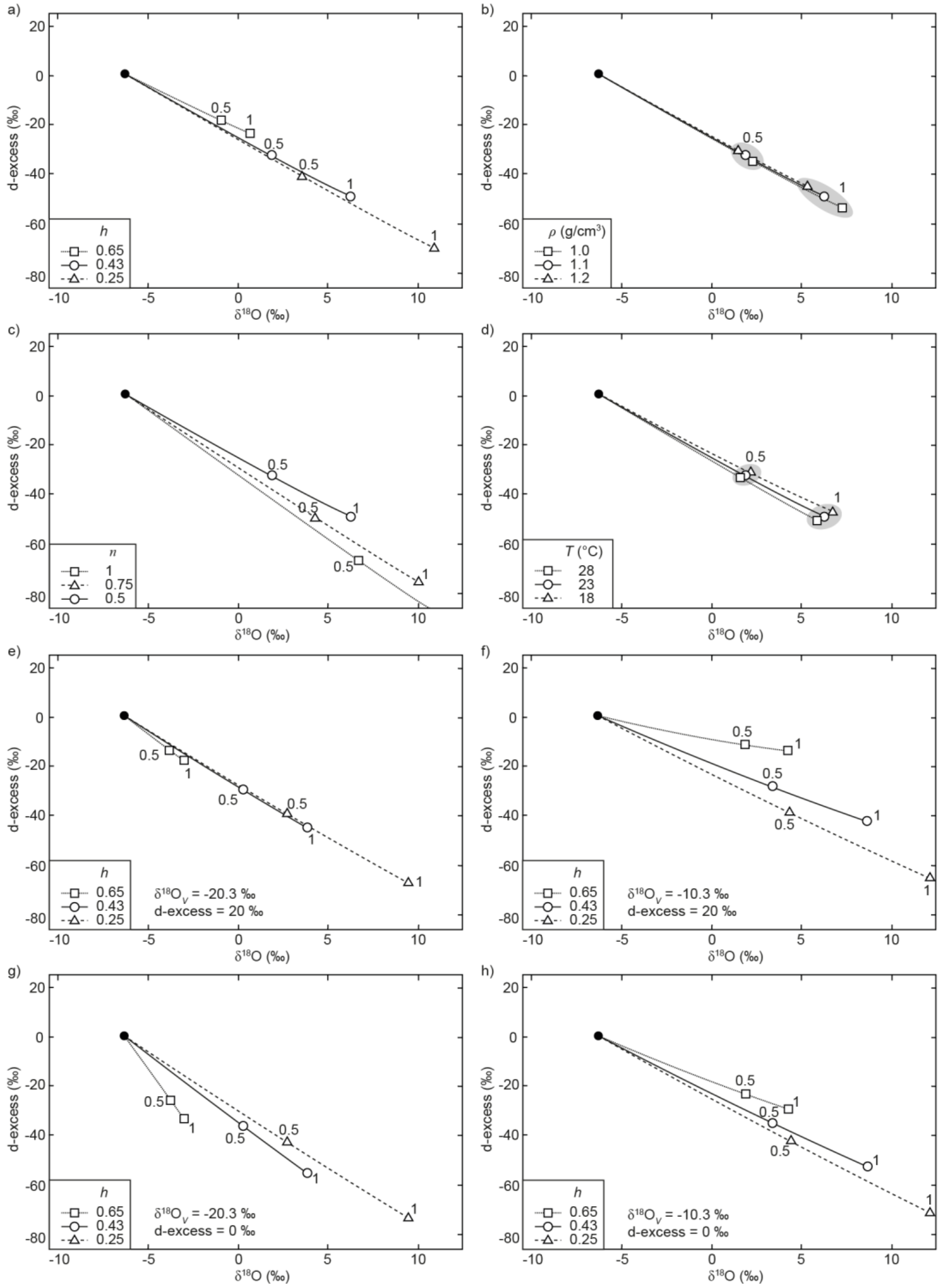

Figure S2: Model sensitivity in d-excess over $\delta^{18}O$ for different variables and parameters during evaporation with recharge of initial water with Salar de Llamara groundwater composition (black dot). Open symbols represent steady states for *E*/*I* of 0.5 and 1 at given *h*, respectively. Normal boundary conditions (solid line) are ρ = 1.1 g/cm^3, T = 23 °C, n = 0.5, h = 0.43, $\delta^{18}O_V$ = -15.3 ‰, and d-excess$_V$ = 10 ‰. **a)** Evaporation at variable relative humidity (h). **b)** Evaporation at variable density (ρ) – proportional to salt content – from which h_{eff} is calculated. **c)** Evaporation at variable wind turbulence, $^*\alpha^n_{l-v\_diff}$. **d)** Evaporation at variable water temperature (T). **e - h)** Evaporation at variable $\delta^{18}O_V$ (-20.3 to -10.3 ‰) and d-excess (0 to 20 per meg).

Supplementary Tables

Table S1. Effect of salinity on ^{17}O-excess measurements (vs. reference gas)

**Salinity** (‰)	**δ'^{17}O** (‰)	**1 σ** (‰)	**δ'^{18}O** (‰)	**1 σ** (‰)	**^{17}O-excess** (per meg)	**1 σ** (per meg)	***n***
0	-3.779	0.025	-7.191	0.042	24	13	3
40	-3.804	0.099	-7.236	0.189	23	10	3
80	-3.815	0.034	-7.247	0.077	18	14	3
120	-3.765	0.141	-7.138	0.261	10	6	3

Table S2. Oxygen and deuterium isotope data (vs. VSMOW) of selected evaporation experiment and pond waters

**Sample**		**δD** (‰)	**± (1 sd)** (‰)	**δ^{18}O** (‰)	**± (1 sd)** (‰)	**d-excess** (‰)	**± (1 sd)** (‰)
		-		-			
exp L0		100.2	0.3	12.7	0.3	1.2	1.0
exp L1		-83.6	0.2	-8.4	0.2	-16.1	0.7
exp L3		-52.6	0.7	-2.0	0.2	-36.9	0.9
exp L4		-44.6	0.8	-1.6	0.5	-32.1	1.6
exp L5		-24.8	0.3	2.8	0.4	-47.2	1.1
pond 1		15.7	0.7	8.7	0.2	-54.0	0.9
	**VI**	**15.7**		**8.7**		**-54.0**	
pond 3		4.9	1.2	7.0	0.0	-51.3	1.2
pond 6		-19.7	1.3	1.3	0.3	-30.1	1.5
pond 7		-30.9	0.1	-1.1	0.2	-22.2	0.4
pond 8		-44.6	0.3	-4.7	0.6	-7.0	1.7
pond 11		-40.6	0.4	-3.2	0.0	-14.9	0.4
	**I**	**-42.6**		**-4.0**		**-11.0**	
site 12 (groundwater)		-49.7	0.2	-6.3	0.3	0.8	0.8

Table S3. Main ion composition of natural water samples and waters from pan evaporation experiments

	**Sample**	**TDS** (g/l)	**ρ** (g/cm^3)	**pH**	**alkalinity** (mmol/kg)	**HCO^{3-}*** (mmol/kg)	**CO_3^{2-}*** (mmol/kg)	**Na^+** (mmol/kg)	**K^+** (mmol/kg)	**Ca^{2+}** (mmol/kg)	**Mg^{2+}** (mmol/kg)	**Cl^-** (mmol/kg)	**SO_4^{2-}** (mmol/kg)
	experiments												
tap water	exp L0	0.2	0.996	7.8	1.65	1.63	0.01	2.52	0.039	0.700	0.012	0.691	0.708
	exp L1	0.2	0.996	7.9	1.91	1.89	0.01	3.22	0.046	0.860	0.021	0.991	0.902
	exp L2	0.3	0.996	7.9	2.05	2.03	0.01	3.56	0.056	0.930	0.037	1.11	0.999
	exp L3	0.3	0.996	8.0	2.55	2.52	0.02	4.53	0.067	1.17	0.033	1.54	1.27
	exp L4	0.4	0.996	8.0	2.70	2.66	0.02	4.83	0.072	1.23	0.029	1.70	1.33
	exp L5	0.4	0.996	8.1	2.49	2.45	0.02	6.39	0.103	1.07	0.041	2.34	1.79
low-TDS water	exp LS0	22.3	1.018	8.0	2.28	2.20	0.04	309	6.11	17.3	5.15	230	58.7
	exp LS1	31.0	1.021	8.2	2.78	2.62	0.08	425	8.53	21.2	7.06	325	80.1
	exp LS2	33.3	1.023	8.2	2.94	2.77	0.08	458	9.12	22.8	7.62	348	86.1
	exp LS3	43.6	1.029	8.3	3.56	3.28	0.14	599	11.9	27.0	9.56	456	111
	exp LS4	43.9	1.030	8.1	3.71	3.52	0.09	601	12.2	25.0	9.76	469	109
	exp LS5	58.5	1.041	8.1	4.53	4.28	0.13	818	17.1	23.2	13.1	632	136
high-TDS water	exp HS0	186	1.126	8.0	3.92	3.65	0.13	2544	54.2	12.4	33.0	1961	355
	exp HS1	213	1.143	8.0	4.39	4.05	0.17	2876	60.7	9.54	36.8	2201	398
	exp HS2	249	1.149	8.1	4.53	4.05	0.24	3350	64.3	9.39	38.8	2606	455
	exp HS3	292	1.180	7.9	5.25	4.85	0.20	3811	76.6	11.5	47.4	3000	511
	exp HS4	286	1.186	7.9	5.43	5.02	0.20	3687	75.5	7.06	46.0	2967	487
	exp HS5	392	1.232	7.8	6.32	5.85	0.24	4911	94.0	5.14	58.0	3911	637
natural waters													
group	pond												
	pond 8	22.5	1.017	8.0	2.29	2.21	0.04	311	6.14	17.3	5.18	231	59.2
	pond 11	16.4	1.009	8.0	2.02	1.96	0.03	192	4.08	19.8	3.92	176	47.0
**I**	**average**	**19.4**	**1.013**	**8.0**	**2.15**	**2.09**	**0.03**	**252**	**5.11**	**18.6**	**4.55**	**203**	**53.1**
**II**	**pond 7**	**32.3**	**1.024**	**8.1**	**2.64**	**2.51**	**0.06**	**451**	**8.85**	**17.7**	**7.17**	**347**	**79.6**
	pond 6	47.7	1.033	8.1	2.81	2.66	0.07	672	13.4	18.4	10.6	520	112
	pond 9	42.7	1.028	8.1	2.61	2.47	0.07	604	11.8	17.7	9.01	475	97.8
	pond 10	38.8	1.028	8.1	2.71	2.58	0.07	540	11.0	18.8	8.63	428	90.6
**III**	**average**	**43.1**	**1.030**	**8.1**	**2.71**	**2.57**	**0.07**	**605**	**12.1**	**18.3**	**9.4**	**474**	**100**
	pond 4	65.7	1.050	8.0	3.16	3.01	0.08	921	21.4	17.3	14.5	750	134
	pond 5	59.4	1.041	8.0	3.04	2.89	0.07	845	17.8	18.1	12.8	659	131
**IV**	**average**	**62.6**	**1.045**	**8.0**	**3.10**	**2.95**	**0.07**	**883**	**19.6**	**17.7**	**13.6**	**704**	**133**
	pond 2	141	1.101	7.9	3.32	3.14	0.09	1968	46.6	14.2	30.0	1542	259
	pond 3	108	1.080	8.0	3.24	3.05	0.10	1505	37.3	16.6	24.3	1240	194
**V**	**average**	**125**	**1.091**	**8.0**	**3.28**	**3.09**	**0.09**	**1737**	**41.9**	**15.4**	**27.1**	**1391**	**226**
**VI**	**pond 1**	**186**	**1.126**	**8.0**	**3.92**	**3.65**	**0.13**	**2544**	**54.2**	**12.4**	**33.0**	**1961**	**355**
	site 12 (groundwater)	4.18	0.999	7.8	1.13	1.12	0.01	24.5	1.02	18.7	1.40	29.0	18.5

* calculated on the basis of pH and alkalinity titration data and SIT model activities (*Grenthe and Wanner*, 2000)

Table S4. *E*/*I* calculations between pond groups based on K^+ and Cl^- concentrations

**Pond group**	**K^+** (mmol/l)	**Cl^-** (mmol/l)	***E*/*I* (K^+)**	***E*/*I* (Cl^-)**	***E*/*I* (ave)**
groundwater	1.02	29			
I	5.17	206	0.80	0.86	0.83
II	9.06	356	0.43	0.42	0.42
III	12.4	488	0.27	0.27	0.27
IV	20.5	736	0.39	0.34	0.36
V	45.7	1517	0.55	0.51	0.53
VI	61.0	2207	0.25	0.31	0.28

Table S5. Pan evaporation model parameters and boundary conditions

**Model step**	***f* (res.)**	**$\delta'^{18}O_{V\_min}$** (‰)	**$\delta'^{18}O_{V\_max}$** (‰)	**wind effect** (*n*)	***T*** (°C)	***h***	***ρ*** (g/cm^3)	**h_{eff}**
tap water								
1	0.82	-16.4	-15.4	0.5	30	0.35	0.996	0.35
2	0.92	-16.4	-15.4	0.5	15	0.75	0.996	0.75
3	0.81	-16.4	-15.4	0.5	30	0.3	0.996	0.30
4	0.95	-16.4	-15.4	0.5	15	0.8	0.996	0.80
5	0.77	-16.4	-15.4	0.5	30	0.3	0.996	0.30
low-TDS water								
1	0.83	-16.4	-15.4	0.5	30	0.35	1.020	0.36
2	0.94	-16.4	-15.4	0.5	15	0.75	1.022	0.77
3	0.81	-16.4	-15.4	0.5	30	0.3	1.026	0.31
4	0.92	-16.4	-15.4	0.5	15	0.8	1.030	0.83
5	0.71	-16.4	-15.4	0.5	30	0.3	1.036	0.31
high-TDS water								
1	0.88	-16.4	-15.4	0.5	30	0.35	1.133	0.40
2	0.91	-16.4	-15.4	0.5	15	0.75	1.146	0.86
3	0.83	-16.4	-15.4	0.5	30	0.3	1.164	0.35
4	0.94	-16.4	-15.4	0.5	15	0.8	1.183	0.95
5	0.8	-16.4	-15.4	0.5	30	0.3	1.209	0.36

Table S6. Recharge model parameters and boundary conditions

**Model step**	***E/I***	**δ′¹⁸O**$_{V\_min}$ (‰)	**δ′¹⁸O**$_{V\_max}$ (‰)	**wind effect** (*n*)	***T*** (°C)	***h***	***ρ*** (g/cm^3)	$h_{eff\_0.4}$	$h_{eff\_0.5}$
1 (groundwater - I)	0.15	-15.8	-14.8	0.5	23	0.4 and 0.5	1.007	0.40	0.50
2 (I - II)	0.15	-15.8	-14.8	0.5	23	0.4 and 0.5	1.021	0.41	0.51
3 (II - III)	0.15	-15.8	-14.8	0.5	23	0.4 and 0.5	1.029	0.41	0.52
4 (II - III)	0.15	-15.8	-14.8	0.5	23	0.4 and 0.5	1.038	0.42	0.52
5 (IV - V)	0.15	-15.8	-14.8	0.5	23	0.4 and 0.5	1.068	0.43	0.53
6 (V - VI)	0.15	-15.8	-14.8	0.5	23	0.4 and 0.5	1.108	0.44	0.56

References

1. Garcés, P. *et al.* Características geoquímicas generales del sistema salino del Salar de Llamara (Chile). *Estud. Geológicos Rev.* **35,** 23–35 (1996).

2. Houston, J. Evaporation in the Atacama Desert: An empirical study of spatio-temporal variations and their causes. *J. Hydrol.* **330,** 402–412 (2006).

3. Bowen, G. J. & Revenaugh, J. Interpolating the isotopic composition of modern meteoric precipitation. *Water Resour. Res.* **39,** 1–13 (2003).

4. Hunt, J. P. & Taube, H. The Exchange of Water Between Hydrated Cations and Solvent. *J. Chem. Phys.* **19,** 602 (1951).

5. Feder, H. M. & Taube, H. Ionic Hydration: An Isotopic Fractionation Technique. *J. Chem. Phys.* **20,** 1335 (1952).

6. Taube, H. Use of oxygen isotope effects in study of hydration of ions. *J. Phys. Chem.* **58,** 523–3654 (1954).

7. Stewart, M. K. & Friedman, I. Deuterium fractionation between aqueous salt solutions and water vapor. *J. Geophys. Res.* **80,** 3812–3818 (1975).

8. Sofer, Z. & Gat, J. R. Activities and concentrations of oxygen-18 in concentrated aqueous salt solutions: Analytical and geophysical implications. *Earth Planet. Sci. Lett.* **15,** 232–238 (1972).

9. Driesner, T. & Seward, T. M. Experimental and simulation study of salt effects and pressure/density effects on oxygen and hydrogen stable isotope liquid-vapor fractionation for 4-5 molal aqueous NaCl and KCl solutions to 400°C. *Geochim. Cosmochim. Acta* **64,** 1773–1784 (2000).

10. Driesner, T., Ha, T.-K. & Seward, T. M. Oxygen and hydrogen isotope fractionation by hydration complexes of Li+, Na+, K+, Mg2+, F-, Cl-, and Br-: A theoretical study. *Geochim. Cosmochim. Acta* **64,** 3007–3033 (2000).

11. Sofer, Z. & Gat, J. R. The isotope composition of evaporating brines: Effect of the isotopic activity ratio in saline solutions. *Earth Planet. Sci. Lett.* **26,** 179–186 (1975).

12. Truesdell, A. H. Oxygen isotope activities and concentrations in aqueous salt solutions at elevated temperatures: Consequences for isotope geochemistry. *Earth Planet. Sci. Lett.* **23,** 387–396 (1974).

13. Koehler, G., Wassenaar, L. I. & Hendry, J. Measurement of stable isotope activities in saline aqueous solutions using optical spectroscopy methods. *Isotopes Environ. Health Stud.* **49,** 378–386 (2013).

14. Criss, R. E. Nonequilibrium Fractionation and Isotopic Transport. in *Principles of Stable Isotope Distribution* (ed. Criss, R. E.) 139–184 (Oxford University Press, 1999).

15. Craig, H. & Gordon, L. Deuterium and oxygen 18 variations in the ocean and the marine atmosphere. in *Stable Isotopes in Oceanographic Studies and Paleotemperatures* (ed. Tongiorgi, E.) 9–130 (Laboratorio die Geologia Nucleare, 1965).

16. Merlivat, L. & Jouzel, J. Global Climatic Interpretation of the Deuterium-Oxygen 18 Relationship for Precipitation. *J. Geophys. Res.* **84,** 5029–5033 (1979).

17. Uemura, R., Barkan, E., Abe, O. & Luz, B. Triple isotope composition of oxygen in atmospheric water vapor. *Geophys. Res. Lett.* **37,** http://dx.doi.org/10.1029/2009GL041960 (2010).

18. Dongmann, G., Nürnberg, H. W., Förstel, H. & Wagener, K. On the Enrichment of $H_2{}^{18}O$ in the Leaves of Transpiring Plants. *Rad. Environ. Biophys.* **11,** 41–52 (1974).

19. Mathieu, R. & Bariac, T. A numerical model for the simulation of stable isotope profiles in drying soils. *J. Geophys. Res.* **101,** 12685–12696 (1996).

20. Haese, B., Werner, M. & Lohmann, G. Stable water isotopes in the coupled atmosphere-land surface model ECHAM5-JSBACH. *Geosci. Model Dev.* **6,** 1463–1480 (2013).
